# 2-(*m*-Tolyl­imino­meth­yl)phenol

**DOI:** 10.1107/S1600536809049307

**Published:** 2009-11-21

**Authors:** Alice Brink, Andreas Roodt, Hendrik G. Visser

**Affiliations:** a Department of Chemistry, University of the Free State, PO Box 339, Bloemfontein 9300, South Africa

## Abstract

The title compound, C_14_H_13_NO, is non-planar with a dihedral angle of 47.00 (6)° between the planes of the two aromatic rings. Intra­molecular hydrogen bonding is observed between the O—H group and the N atom, resulting in a phenol–imine tautomeric form.

## Related literature

For related structures, see: Elmali *et al.* (1998 ▶); Cheng *et al.* (2005 ▶); Arod *et al.* (2005 ▶); Bingöl *et al.* (2007 ▶); Zhang *et al.* (2007 ▶). For macromolecules containing Schiff base–metal complexes see: Leung *et al.* (2007 ▶). For related structures with non-linear optical properties, magnetic, oxygen transport and catalytic properties: see Karakas *et al.* (2004 ▶); Miyasaka *et al.* (2003 ▶); Bailes *et al.* (1947 ▶); Zhang *et al.* (1994 ▶). For photo-physical properties such as thermochromism and photochromism, see: Gakias *et al.* (2005 ▶). For *N*-Salicylideneaniline, which displays reversible photoreactivity and crystallizes as both non-planar and planar polymorphs, see: Arod *et al.* (2005 ▶, 2007 ▶)
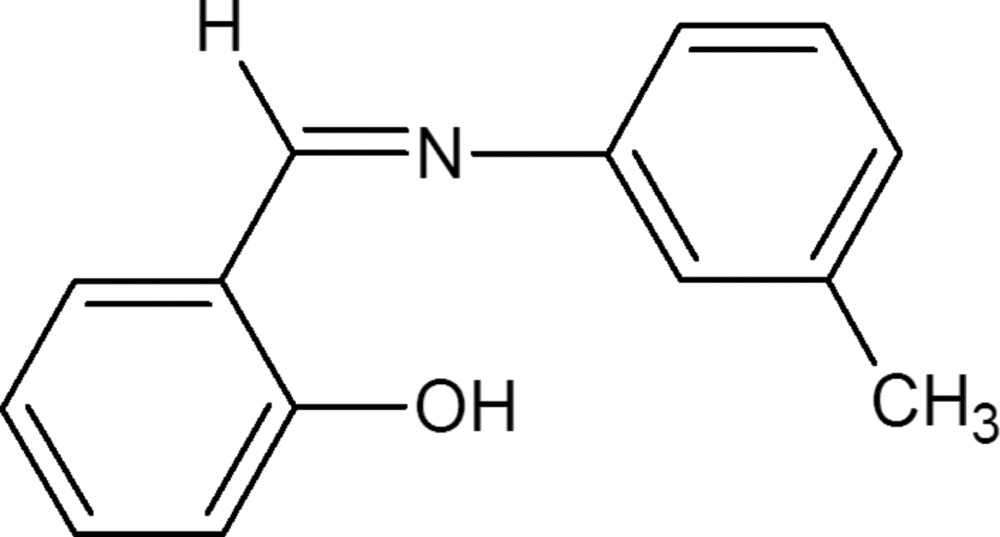


## Experimental

### 

#### Crystal data

C_14_H_13_NO
*M* *_r_* = 211.25
Orthorhombic, 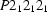

*a* = 7.4946 (4) Å
*b* = 11.8669 (6) Å
*c* = 12.2970 (6) Å
*V* = 1093.67 (10) Å^3^
*Z* = 4
Mo *K*α radiation
μ = 0.08 mm^−1^
*T* = 100 K
0.35 × 0.22 × 0.08 mm

#### Data collection

Bruker X8 APEXII 4K Kappa CCD diffractometer
Absorption correction: multi-scan (*SADABS*; Bruker, 2004 ▶) *T*_min_ = 0.972, *T*_max_ = 0.994
11186 measured reflections
1532 independent reflections
1210 reflections with *I* > 2σ(*I*)
*R*_int_ = 0.055

#### Refinement

*R*[*F* ^2^ > 2σ(*F* ^2^)] = 0.043
*wR*(*F* ^2^) = 0.099
*S* = 1.08
1532 reflections
146 parameters
H-atom parameters constrained
Δρ_max_ = 0.20 e Å^−3^
Δρ_min_ = −0.21 e Å^−3^

### 

Data collection: *APEX2* (Bruker, 2005 ▶); cell refinement: *SAINT-Plus* (Bruker, 2004 ▶); data reduction: *SAINT-Plus* and *XPREP* (Bruker, 2004 ▶); program(s) used to solve structure: *SIR92* (Altomare *et al.*, 1999 ▶); program(s) used to refine structure: *SHELXL97* (Sheldrick, 2008 ▶); molecular graphics: *DIAMOND* (Brandenburg & Putz, 2004 ▶); software used to prepare material for publication: *WinGX* (Farrugia, 1999 ▶).

## Supplementary Material

Crystal structure: contains datablocks global, I. DOI: 10.1107/S1600536809049307/bg2304sup1.cif

Structure factors: contains datablocks I. DOI: 10.1107/S1600536809049307/bg2304Isup2.hkl

Additional supplementary materials:  crystallographic information; 3D view; checkCIF report

## Figures and Tables

**Table 1:** Hydrogen-bond geometry (Å, °)

*D*—H⋯*A*	*D*—H	H⋯*A*	*D*⋯*A*	*D*—H⋯*A*
O1—H1*A*⋯N1	0.84	1.85	2.595 (2)	147

**Fig. 1.**
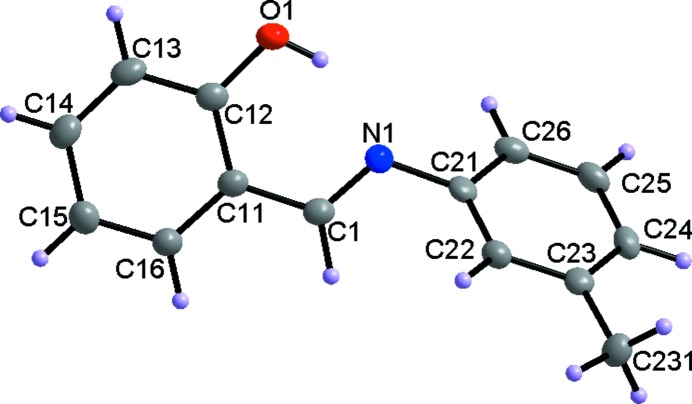
Molecular structure of the title compound. Displacement ellipsoids are drawn at the 50% probability level.

## supplementary crystallographic information

### Comment

Schiff bases readily form stable complexes with many transition metals and therefore play an important role in the developement of coordination chemistry. The choice of the appropriate amine and various substituents on the aromatic ring of the carbonyl compound allows for great versatility and fine tuning of the steric and electronic properties. The ligands have interesting photo-physical properties as thermochromism and photochromism (Gakias *et al.*, 2005), such as N-Salicylideneaniline which displays reversible photoreactivity and crystallizes as both non-planar and planar polymorphs (Arod *et al.*, 2005 and 2007).

The title compound (Figure 1) is non-planar with a dihedral angle between aromatic rings of 47.00 (6)°, and a C1—N1—C21—C22 torsion angle of 42.7 (3)° . The C1—N1 bond distance (1.287 (3) Å) confirms to the value for a double bond. The molecule displays a *trans* configuration with respect to the C1—N1 double bond. The N1—C21 and C1—C11 bond distances are 1.426 (3) and 1.445 (3)Å respectively and all other bond distances are within normal range. The molecule adopts a phenol-imine tautomeric form, with strong intramolecular hydrogen bonding observed (O—H···N = 2.595 (2) Å).

### Experimental

Salicylaldehyde (14.0 mmol, 1.71 g) was dissolved in 30 ml methanol. *m*-Toluidine (14.0 mmol, 1.50 g) dissolved in methanol (10 ml), was added dropwise to the reaction mixture. Anhydrous MgSO_4_ was added to the reaction. The mixture was heated to 80° and refluxed for 3 hr. The MgSO_4_ was filtered and the yellow product was collected. The solvent was removed under reduced pressure. The product was dissolved in acetone and crystals suitable for *x*-ray diffraction were obtained by slow evaporation of the solvent at 0°C. Yield 94.4%. ^1^H NMR [CDCl_3_, 300 MHz, δ(p.p.m.)] 8.6 (1*H*, s, HCN), 7.4–6.9 (8*H*, m, Ar), 2.4 (3*H*, s, CH_3_).

### Refinement

H atoms were placed in geometrically idealized positions and constrained to ride on their parent atoms, with C—H = 0.95-0.98 Å, O—H: 0.84 Å and *U*_iso_(H) = 1.2-1.5 *U*_eq_(C). The methyl groups were generated to fit the difference electron density and the groups were then refined as rigid rotors. In the absence of significant anomalous scattering effects Friedel pairs have been merged.

### Crystal data

**Table:**

C_14_H_13_NO	*F*(000) = 448
*M**_r_* = 211.25	*D*_x_ = 1.283 Mg m^−^^3^
Orthorhombic, *P*2_1_2_1_2_1_	Mo *K*α radiation, λ = 0.71073 Å
Hall symbol: P 2ac 2ab	Cell parameters from 1942 reflections
*a* = 7.4946 (4) Å	θ = 3.2–22.3°
*b* = 11.8669 (6) Å	µ = 0.08 mm^−^^1^
*c* = 12.2970 (6) Å	*T* = 100 K
*V* = 1093.67 (10) Å^3^	Cuboid, red
*Z* = 4	0.35 × 0.22 × 0.08 mm

### Data collection

**Table:**

Bruker X8 APEXII 4K Kappa CCD diffractometer	1532 independent reflections
Radiation source: sealed tube	1210 reflections with *I* > 2σ(*I*)
graphite	*R*_int_ = 0.055
Detector resolution: 512 pixels mm^-1^	θ_max_ = 28.0°, θ_min_ = 2.4°
ω and φ scans	*h* = −9→9
Absorption correction: multi-scan (*SADABS*; Bruker, 2004)	*k* = −15→12
*T*_min_ = 0.972, *T*_max_ = 0.994	*l* = −13→16
11186 measured reflections	

### Refinement

**Table:**

Refinement on *F*^2^	0 restraints
Least-squares matrix: full	H-atom parameters constrained
*R*[*F*^2^ > 2σ(*F*^2^)] = 0.043	*w* = 1/[σ^2^(*F*_o_^2^) + (0.0482*P*)^2^ + 0.0939*P*] where *P* = (*F*_o_^2^ + 2*F*_c_^2^)/3
*wR*(*F*^2^) = 0.099	(Δ/σ)_max_ < 0.001
*S* = 1.08	Δρ_max_ = 0.20 e Å^−^^3^
1532 reflections	Δρ_min_ = −0.21 e Å^−^^3^
146 parameters	

### Special details

**Table:**

Experimental. The intensity data was collected on a Bruker X8 Apex II 4 K Kappa CCD diffractometer using an exposure time of 60 s/frame. A total of 1032 frames were collected with a frame width of 0.5° covering up to θ = 27.99° with 100.0% completeness accomplized.
Geometry. All e.s.d.'s (except the e.s.d. in the dihedral angle between two l.s. planes) are estimated using the full covariance matrix. The cell e.s.d.'s are taken into account individually in the estimation of e.s.d.'s in distances, angles and torsion angles; correlations between e.s.d.'s in cell parameters are only used when they are defined by crystal symmetry. An approximate (isotropic) treatment of cell e.s.d.'s is used for estimating e.s.d.'s involving l.s. planes.

### Fractional atomic coordinates and isotropic or equivalent isotropic displacement parameters (Å^2^)

**Table:**

	*x*	*y*	*z*	*U*_iso_*/*U*_eq_	
C1	0.4954 (3)	0.32440 (17)	0.70887 (17)	0.0219 (5)	
H1	0.4568	0.2514	0.6877	0.026*	
C231	0.5244 (3)	0.15389 (17)	0.30941 (18)	0.0272 (5)	
H2A	0.584	0.1037	0.3612	0.041*	
H2B	0.6073	0.1733	0.2507	0.041*	
H2C	0.4198	0.1158	0.2789	0.041*	
C11	0.5390 (3)	0.34554 (16)	0.82153 (17)	0.0209 (5)	
C12	0.6066 (3)	0.45064 (17)	0.85663 (18)	0.0223 (5)	
C13	0.6401 (3)	0.46927 (19)	0.96612 (18)	0.0257 (5)	
H13	0.6882	0.5393	0.9893	0.031*	
C14	0.6037 (3)	0.38626 (18)	1.0414 (2)	0.0281 (5)	
H14	0.6248	0.4004	1.1163	0.034*	
C15	0.5364 (3)	0.28196 (18)	1.00924 (18)	0.0290 (6)	
H15	0.5114	0.2252	1.0616	0.035*	
C16	0.5066 (3)	0.26242 (17)	0.90006 (17)	0.0242 (5)	
H16	0.4631	0.1909	0.8776	0.029*	
C21	0.4611 (3)	0.37863 (16)	0.52673 (17)	0.0207 (5)	
C22	0.5118 (3)	0.27911 (17)	0.47524 (17)	0.0214 (5)	
H22	0.5779	0.224	0.5142	0.026*	
C23	0.4662 (3)	0.25991 (17)	0.36708 (18)	0.0214 (5)	
C24	0.3690 (3)	0.34139 (17)	0.31147 (18)	0.0237 (5)	
H24	0.3362	0.329	0.2378	0.028*	
C25	0.3195 (3)	0.44076 (17)	0.36278 (19)	0.0258 (5)	
H25	0.2525	0.4956	0.3241	0.031*	
C26	0.3668 (3)	0.46058 (17)	0.46985 (18)	0.0240 (5)	
H26	0.3351	0.5295	0.5042	0.029*	
N1	0.5078 (2)	0.40260 (13)	0.63674 (14)	0.0213 (4)	
O1	0.6371 (2)	0.53517 (12)	0.78487 (12)	0.0274 (4)	
H1A	0.6018	0.5156	0.7228	0.041*	

### Atomic displacement parameters (Å^2^)

**Table:**

	*U*^11^	*U*^22^	*U*^33^	*U*^12^	*U*^13^	*U*^23^
C1	0.0191 (12)	0.0190 (10)	0.0276 (12)	0.0004 (9)	0.0021 (11)	−0.0022 (9)
C231	0.0276 (13)	0.0287 (11)	0.0253 (11)	0.0035 (10)	−0.0034 (11)	−0.0028 (10)
C11	0.0200 (11)	0.0176 (10)	0.0251 (11)	0.0030 (9)	0.0013 (10)	−0.0020 (9)
C12	0.0176 (11)	0.0205 (11)	0.0287 (13)	0.0023 (9)	0.0018 (10)	−0.0013 (10)
C13	0.0202 (12)	0.0232 (10)	0.0338 (13)	−0.0003 (10)	−0.0021 (11)	−0.0064 (10)
C14	0.0272 (13)	0.0303 (12)	0.0267 (12)	0.0029 (11)	−0.0032 (11)	−0.0054 (10)
C15	0.0341 (14)	0.0269 (12)	0.0260 (12)	0.0025 (11)	0.0013 (11)	0.0023 (10)
C16	0.0276 (13)	0.0208 (10)	0.0242 (12)	0.0002 (10)	0.0021 (10)	−0.0015 (8)
C21	0.0174 (11)	0.0218 (10)	0.0227 (12)	−0.0051 (9)	0.0036 (10)	0.0035 (9)
C22	0.0191 (11)	0.0213 (10)	0.0238 (12)	0.0013 (9)	0.0002 (10)	0.0031 (9)
C23	0.0161 (11)	0.0235 (10)	0.0247 (11)	−0.0032 (9)	0.0010 (10)	0.0018 (9)
C24	0.0182 (11)	0.0325 (12)	0.0203 (11)	−0.0023 (10)	0.0003 (10)	0.0060 (10)
C25	0.0222 (12)	0.0245 (12)	0.0305 (13)	0.0001 (9)	0.0006 (11)	0.0100 (10)
C26	0.0202 (12)	0.0187 (10)	0.0331 (13)	0.0002 (10)	0.0029 (11)	0.0032 (10)
N1	0.0208 (10)	0.0213 (9)	0.0219 (9)	0.0016 (8)	0.0019 (9)	0.0001 (7)
O1	0.0315 (9)	0.0207 (7)	0.0300 (8)	−0.0036 (7)	−0.0004 (8)	−0.0007 (7)

### Geometric parameters (Å, °)

**Table:**

C1—N1	1.287 (3)	C15—C16	1.381 (3)
C1—C11	1.445 (3)	C15—H15	0.95
C1—H1	0.95	C16—H16	0.95
C231—C23	1.509 (3)	C21—C26	1.391 (3)
C231—H2A	0.98	C21—C22	1.393 (3)
C231—H2B	0.98	C21—N1	1.426 (3)
C231—H2C	0.98	C22—C23	1.392 (3)
C11—C16	1.402 (3)	C22—H22	0.95
C11—C12	1.414 (3)	C23—C24	1.390 (3)
C12—O1	1.355 (2)	C24—C25	1.388 (3)
C12—C13	1.387 (3)	C24—H24	0.95
C13—C14	1.379 (3)	C25—C26	1.384 (3)
C13—H13	0.95	C25—H25	0.95
C14—C15	1.394 (3)	C26—H26	0.95
C14—H14	0.95	O1—H1A	0.84
			
N1—C1—C11	121.27 (18)	C15—C16—C11	121.6 (2)
N1—C1—H1	119.4	C15—C16—H16	119.2
C11—C1—H1	119.4	C11—C16—H16	119.2
C23—C231—H2A	109.5	C26—C21—C22	120.19 (19)
C23—C231—H2B	109.5	C26—C21—N1	117.55 (18)
H2A—C231—H2B	109.5	C22—C21—N1	122.22 (18)
C23—C231—H2C	109.5	C23—C22—C21	120.40 (19)
H2A—C231—H2C	109.5	C23—C22—H22	119.8
H2B—C231—H2C	109.5	C21—C22—H22	119.8
C16—C11—C12	118.21 (19)	C24—C23—C22	119.0 (2)
C16—C11—C1	119.93 (18)	C24—C23—C231	120.03 (19)
C12—C11—C1	121.80 (18)	C22—C23—C231	121.0 (2)
O1—C12—C13	118.91 (19)	C25—C24—C23	120.5 (2)
O1—C12—C11	120.96 (19)	C25—C24—H24	119.8
C13—C12—C11	120.1 (2)	C23—C24—H24	119.8
C14—C13—C12	120.1 (2)	C26—C25—C24	120.6 (2)
C14—C13—H13	119.9	C26—C25—H25	119.7
C12—C13—H13	119.9	C24—C25—H25	119.7
C13—C14—C15	121.0 (2)	C25—C26—C21	119.3 (2)
C13—C14—H14	119.5	C25—C26—H26	120.3
C15—C14—H14	119.5	C21—C26—H26	120.3
C16—C15—C14	118.9 (2)	C1—N1—C21	119.49 (16)
C16—C15—H15	120.5	C12—O1—H1A	109.5
C14—C15—H15	120.5		
			
N1—C1—C11—C16	173.3 (2)	C26—C21—C22—C23	−1.1 (3)
N1—C1—C11—C12	−3.6 (3)	N1—C21—C22—C23	−178.81 (19)
C16—C11—C12—O1	−178.59 (19)	C21—C22—C23—C24	−0.2 (3)
C1—C11—C12—O1	−1.6 (3)	C21—C22—C23—C231	178.35 (19)
C16—C11—C12—C13	0.4 (3)	C22—C23—C24—C25	0.5 (3)
C1—C11—C12—C13	177.4 (2)	C231—C23—C24—C25	−178.0 (2)
O1—C12—C13—C14	177.47 (19)	C23—C24—C25—C26	0.3 (3)
C11—C12—C13—C14	−1.5 (3)	C24—C25—C26—C21	−1.5 (3)
C12—C13—C14—C15	1.3 (3)	C22—C21—C26—C25	1.9 (3)
C13—C14—C15—C16	0.1 (4)	N1—C21—C26—C25	179.75 (19)
C14—C15—C16—C11	−1.3 (4)	C11—C1—N1—C21	−179.12 (19)
C12—C11—C16—C15	1.0 (3)	C26—C21—N1—C1	139.4 (2)
C1—C11—C16—C15	−176.0 (2)	C22—C21—N1—C1	−42.8 (3)

### Hydrogen-bond geometry (Å, °)

**Table:**

*D*—H···*A*	*D*—H	H···*A*	*D*···*A*	*D*—H···*A*
O1—H1A···N1	0.84	1.85	2.595 (2)	147
